# Adenosine triphosphate-functionalized graphene oxide for selective recovery of Nd(iii) and Y(iii): synthesis, mechanism, and performance evaluation

**DOI:** 10.1039/d6ra02358d

**Published:** 2026-07-02

**Authors:** Effat A. Jebril, Mohamed M. Hefny, Mohamed S. El-Deab, Walaa S. Hafez, Mohamed A. M. Youssef, Taysseer A. Lasheen

**Affiliations:** a Nuclear Materials Authority P.O. Box 530, El Maadi Cairo Egypt Effat.nma@gmail.com; b Chemistry Department, Faculty of Science, Cairo University Cairo Egypt

## Abstract

The development of bio-functionalized nanomaterials provides an effective pathway toward sustainable recovery of rare earth elements (REEs) from aqueous systems. In this work, adenosine triphosphate-functionalized graphene oxide (GO-ATP) was synthesized through covalent immobilization of ATP onto GO nanosheets, generating a multifunctional interfacial surface enriched with phosphate and hydroxyl donor groups. Comprehensive characterization using FTIR, SEM/EDX, BET, XRD, and TEM confirmed successful ATP grafting, increased surface heterogeneity, and the preservation of structural integrity. Batch adsorption experiments demonstrated high affinities toward Nd^3+^ and Y^3+^, with rapid equilibrium attainment and excellent correlation with the pseudo-second-order kinetic model, indicating chemisorption controlled uptake. The Langmuir isotherm adequately described the sorption behavior, suggesting monolayer adsorption, while thermodynamic parameters revealed a spontaneous and endothermic process. Mechanistic interpretation based on HSAB theory and surface complexation concepts showed that REE ions coordinate strongly with phosphate oxygen and nucleophilic nitrogen sites of ATP, forming stable inner-sphere complexes. The GO-ATP adsorbent also exhibited remarkable selectivity and reusability, maintaining >90% removal efficiency over repeated cycles. These findings highlight GO-ATP as a green, biocompatible, and highly selective platform for REE recovery and advanced water purification applications.

## Abstract 2

The development of bio-functionalized nanomaterials provides an effective pathway toward sustainable recovery of rare earth elements (REEs) from aqueous systems.

## Graphical abstract

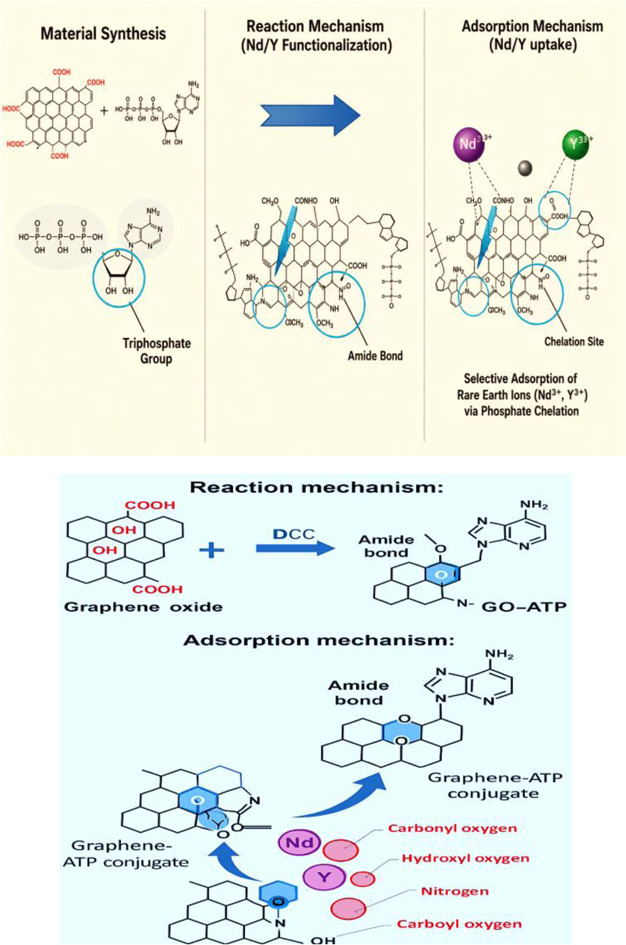

## 1. Introduction

Rare Earth Elements (REEs), a group of 17 metallic elements, are indispensable strategic resources for modern technological industries and everyday consumer goods. Among these, Neodymium (Nd) and Yttrium (Y) hold particular importance. Nd is a crucial component in high strength permanent magnets, which are vital for the manufacturing of electric vehicles and wind turbines, playing a critical role in the global transition towards a low carbon and renewable energy society. Yttrium, alongside other REEs, finds extensive applications in electronics, catalysts, luminescent materials, and hydrogen storage technologies.^1–3^ The unique magnetic, luminescent, and electrical properties of these elements have driven a steady increase in their global demand over recent decades.^4,5^ The escalating demand for REEs, coupled with the severe environmental consequences of primary extraction and the inherent risks of a concentrated supply chain, creates a compelling imperative for developing sustainable recovery methods from secondary resources. This shift is not merely an environmental consideration; it represents a strategic imperative for global technological independence and responsible environmental stewardship.^6^

Among the rare earth elements investigated in this study, Nd^3+^ and Y^3+^ were selected as representative models of light rare earth elements (LREEs) and heavy rare earth elements (HREEs, yttrium group), respectively. In addition to their strategic technological importance, both elements commonly coexist in Egyptian monazite-derived raffinate solutions. Their selection therefore provides a practical basis for evaluating the adsorption performance and selectivity of the developed adsorbent toward representative REE classes.

Various conventional methods have been employed for the separation and recovery of REEs from aqueous solutions, including solvent extraction, electrochemical extraction, ion exchange, resin separation, and chemical precipitation under specific conditions.^7^ In this context, adsorption has emerged as a particularly promising and alternative method has been used for the REEs separation from unconventional aqueous sources. Its widespread adoption is attributed to several significant advantages, including environmental friendliness, tunable selectivity, simplicity of operation, low cost, high efficiency, minimal waste generation, reduced reliance on organic solvents, and rapid phase separation.^7–11^ The effectiveness of adsorption, however, is highly contingent upon the performance characteristics of the adsorbent material itself. Ideal adsorbent materials should possess a substantial specific surface area and abundant surface functional groups to facilitate effective and selective binding of REE ions.^7^ The development of superior adsorbent materials is thus a core challenge and opportunity in this field. Graphene-based materials, particularly graphene oxide (GO), have garnered significant attention in environmental pollution treatment due to their exceptional properties. GO possesses a unique two-dimensional structure, a high theoretical specific surface area, excellent electrical and thermal conductivity, and superior mechanical strength.^12^ As an oxidized derivative of graphene, GO is characterized by an abundance of oxygen-containing functional groups, including carboxyl (–COOH), hydroxyl (–OH), carbonyl (C=O), and epoxy groups, distributed across its basal planes and edges.^13^ These functional groups provide numerous active sites for pollutant adsorption and contribute to GO intrinsic hydrophilicity and dispersibility in aqueous media. GO has been extensively investigated and shown to be highly effective for the removal of various heavy metals and rare earth ions from aqueous solutions.^13^

The primary interaction mechanisms involved in adsorption on graphene-based materials typically include π–π interaction, hydrogen bonding, and electrostatic interaction.^14^ Despite its remarkable intrinsic adsorption capacity, the practical application of GO is often hindered by certain limitations. Its high hydrophilicity and good dispersibility, while beneficial for initial interaction, can lead to poor recyclability and reusability, making solid–liquid separation challenging. Furthermore, GO sheets tend to self-agglomerate, which can significantly reduce their effective surface area and limit the accessibility of active sites.^13^ These challenges highlight a critical design principle for advanced adsorbents: combining high adsorption performance with practical considerations like recoverability and reusability. Therefore, functionalized modification of GO is crucial to retain its beneficial properties while overcoming these limitations, ultimately improving its adsorption properties, selectivity, and ease of separation from treated water.

The strong affinity of phosphate-containing compounds for REEs is a well-established phenomenon, offering greater selectivity towards REEs over other common competing cations such as Na^+^, Mg^2+^, and Fe^3+^.^6^ Modified graphene oxide with phosphate groups has been shown to significantly enhance its adsorption capacity and selectivity for various metal ions and other REEs.^15^ For instance, phosphate-functionalized graphene oxide (PGO), prepared by grafting triethyl phosphite onto the GO surface, demonstrated a high adsorption capacity for U(vi), which is substantially higher than that of pristine GO. This enhancement is consistently attributed to the direct coordination of the phosphate group with the metal ions, as confirmed by spectroscopic analyses like EDX and FTIR after adsorption.^16^ Other modification strategies have further validated this concept. Al-Salem *et al.*^13^ reported the fabrication of magnetic chitosan–graphene oxide composites for the adsorption of neodymium, demonstrating high uptake capacity due to the synergistic contribution of graphene oxide's layered structure and chitosan's abundant functional groups. Similarly, Zhao *et al.*^17^ synthesized graphene oxide–tris(4-aminophenyl)amine composites and revealed that the introduction of amine groups enhanced the interaction between rare earth cations and oxygen/nitrogen donor atoms, confirming that surface modification of graphene oxide significantly improves REE binding performance. In another study, Ghaly *et al.*^18^ prepared magnesium oxide-calcium alginate hydrogels as hybrid adsorbents for yttrium and neodymium removal, where the incorporation of MgO nanoparticles into the alginate matrix generated additional active sites and improved mechanical stability. Although direct literature on GO modified by adenosine triphosphate (ATP) specifically for Nd and Y removal is limited, ATP is a nucleotide molecule inherently rich in multiple phosphate groups. Therefore, the established principles and advantages observed for general phosphate-functionalized graphene oxide (PGO) in REE adsorption are highly relevant and directly applicable to a GO-ATP nanocomposite. Given the strong and selective affinity of phosphate groups for REEs, and the proven enhancement of GO adsorption capabilities upon phosphate functionalization, it is hypothesized that modifying GO with ATP would similarly create a highly effective adsorbent for Nd and Y.

This study aims to comprehensively investigate the synthesis, characterization, and adsorption performance of a graphene oxide modified by adenosine triphosphate (GO-ATP) nanocomposite for the efficient removal of Neodymium (Nd) and Yttrium (Y) from aqueous solutions. The study will delve into the kinetics, isotherm, and thermodynamic aspects of the adsorption process to gain a fundamental understanding of the interactions between the REE ions and the novel adsorbent. Furthermore, a detailed mechanism of adsorption will be proposed, integrating the roles of various functional groups and interaction types. The findings from this research are expected to contribute significantly to the development of advanced, sustainable materials for REE recovery, addressing critical environmental and resource challenges.

## 2. Materials and methods

### 2.1 Materials

Graphite powder (purity >99%), potassium permanganate (KMnO_4_), sulfuric acid (H_2_SO_4_), hydrogen peroxide (H_2_O_2_, 30%), hydrochloric acid (HCl), adenosine triphosphate (ATP, disodium salt hydrate), dicyclohexyl carbodiimide (DCC), dimethyl formamide (DMF), neodymium nitrate (Nd(NO_3_)_3_·6H_2_O), and yttrium nitrate (Y(NO_3_)_3_·6H_2_O) were obtained from Sigma-Aldrich and used without further purification. Double distilled water (DDW) was used for all solution preparations.

### 2.2 Synthesis of graphene oxide

Graphene oxide GO was synthesized *via* a modified Hummers' method,^19,20^ involving the chemical oxidation of natural graphite flakes using potent oxidizing agents, including sulfuric acid (H_2_SO_4_) and potassium permanganate (KMnO_4_). The detailed procedure is as follows: a mixture of 2 g graphite and 2 g sodium nitrate (NaNO_3_) was combined with 92 mL of concentrated H_2_SO_4_ under constant stirring. 12 g of KMnO_4_ was gradually added to the flask while maintaining the reaction in an ice-water bath. The mixture was agitated for 2 hours before transferring the flask to a 35–40 °C water bath, where stirring continued for an additional 4 hours. Subsequently, 200 mL of double distilled water was introduced, and the suspension was held at 98 °C for 30 minutes. To terminate oxidation, 20 mL of 30% hydrogen peroxide (H_2_O_2_) was added, inducing a color shift from brown to yellow. The resulting product was centrifuged at 7000 rpm for 15 minutes, and the solid phase was subjected to three washing cycles with 0.1 M hydrochloric acid (HCl). Residual acids were removed by rinsing the sample repeatedly with double distilled water until a neutral pH was achieved. Finally, the purified GO was exfoliated *via* intense ultra-sonication and dried under vacuum at 40 °C.

### 2.3 Synthesis of GO-ATP nanocomposite

**Fig. 1:**
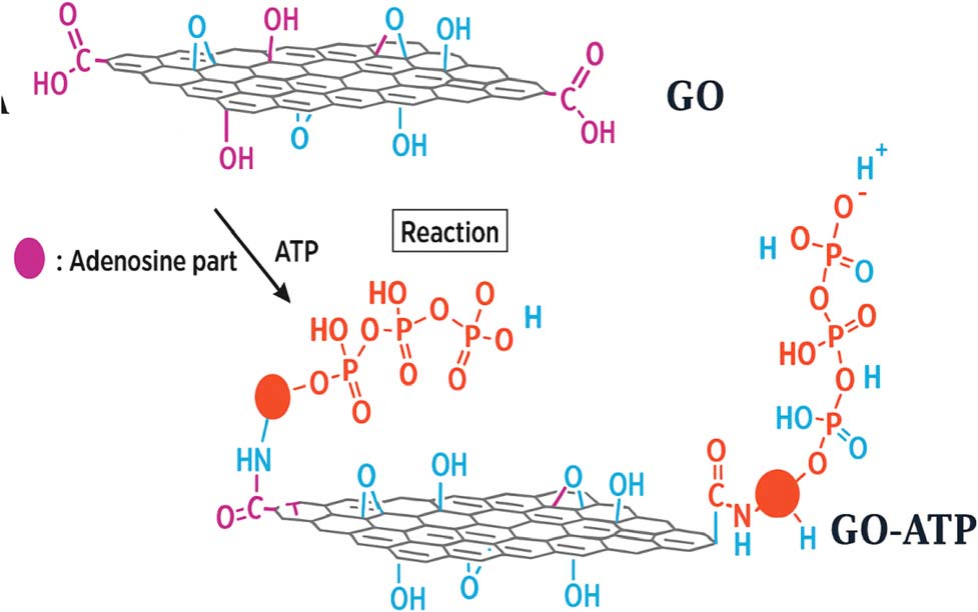
A schematic diagram showing the synthesis of GO-ATP adsorbent.

To prepare the GO-ATP nanocomposite, 0.4 g of synthesized GO was dispersed in 14 mL of DMF *via* ultrasonication for 1 hour. Then add 0.4 g of ATP and 0.4 g of DCC to the above solution. The mixture was refluxed in around bottom flask for 24 h at 70 °C. After refluxing, the content in the flask was filtered through a Buchner funnel fitted with Whatman filter paper no. 41. The as-obtained black coloured residue was washed with hot double distilled water and was left for overnight oven drying at 60 °C, Fig. 1.^21^

### 2.4 Characterization techniques

Thermostat bath (GFL 1083, Germany), and a digital pH meter (Digimed DM-21, UK) was used to measure pH. The UV/Vis spectrophotometer of type (Labomed, Inc. U.S.A.) was used to determine the elements by arsenazo III method. Micrographs and elemental analysis of GO and GO-ATP were obtained by SEM and EDX (Prisma E (Thermo Scientific), USA). A MICROTRAC MRB (BELSORP MINI X) surface area and porosity analyzer was used to calculate the surface area, pore diameter, and pore volume using the BET and BJH methods from the N_2_ sorption isotherm. The FT-IR infrared spectrum was acquired using (Thermo Scientific, NICOLET iS10, USA). XRD, Melvern Panalytical Empryan2020 device model, Netherlands. Cu tube operated at 40 kV and 30 mA. and using Ni filter. Identify the phases constituting, according PDF-2 cards release 2020, the synthesis diagram of GO-ATP materials is shown in Fig. 1. Zeta potential measurements were performed using a NICOMP 380 ZLS instrument (Particle Sizing Systems, Santa Barbara, CA, USA). The system employed a He–Ne laser (*λ* = 632 nm) with a detection angle of 18.9° for electrophoretic mobility analysis. Experiments were conducted at the Nanomaterial Investigation Laboratory, Central Laboratory Network (CLN), and National Research Centre (NRC). For sample preparation, 0.2 g of the composite material was dispersed in 5 mL of Millipore water and ultrasonicated for 5 min to obtain a homogeneous suspension before measurement.

### 2.5 Batch adsorption experiments

Batch adsorption studies were performed to evaluate the removal efficiency of Nd^3+^ and Y^3+^. Adsorption parameters such as contact time (5–300 min), initial ion concentration (10–300 mg L^−1^), pH (1–6), and temperature (25–55 °C) were systematically varied. A fixed dose of GO-ATP (20 mg) was added to 25 mL of metal ion solution in 50 mL conical flasks, which were agitated at 230 rpm in a thermostatic shaker. The samples were filtered, and the residual metal concentration was analyzed using UV-visible double beam spectrophotometer model (Labomed, Inc. U. S. A.) and ICP-OES (Teledyne Leeman labs, U. S. A.). All experiments were conducted in triplicate, and average values were reported. The adsorption capacity (mg g^−1^) at time and the equilibrium adsorption capacity were calculated using the following equations:1
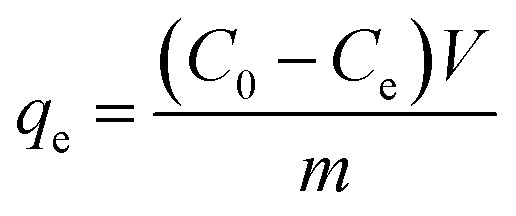
2
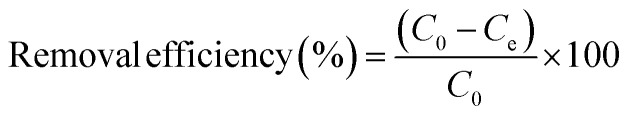
where: *q*_e_ is the adsorption capacity, *C*_0_ and *C*_e_ = initial and equilibrium REES concentrations (mg L^−1^), *V* = solution volume (L), *m* = mass of adsorbent (g). These values were further analyzed to model adsorption isotherms and kinetics.

### 2.6 Regeneration study and desorption of adsorbed REEs

After the adsorption experiments, the spent adsorbent was rinsed thoroughly with water and left to dry overnight. Subsequently, it was treated with 25 mL of hydrochloric acid (HCl), sulfuric acid (H_2_SO_4_), or nitric acid (HNO_3_) solutions at varying concentrations. Once equilibrium was reached, the mixture was filtered, and the concentrations of Nd^3+^, and Y^3+^ ions in the filtrate were determined. The regenerated adsorbent was then washed repeatedly with double-distilled water to neutralize any residual acid and was prepared for reuse in further adsorption cycles. The adsorption–desorption process was repeated over five consecutive cycles under optimal conditions. The desorption efficiency was calculated using eqn (3):3
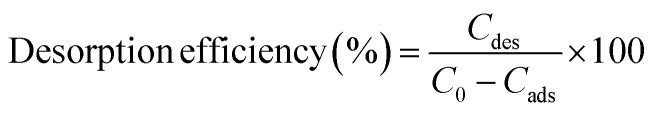
where *C*_0_ is initial concentrations (mg L^−1^), *C*_des_ is the desorption concentration (mg L^−1^), while *C*_ads_ is the solution concentration after adsorption (mg L^−1^).

### 2.7 Application to real raffinate solution

The practical applicability of GO-ATP was evaluated using a real raffinate solution obtained from the hydrometallurgical processing of Egyptian monazite ore. Batch adsorption tests were performed by contacting 0.02 g of GO-ATP with 25 mL of raffinate at optimum conditions under constant shaking. After adsorption, the mixture was filtered and the residual ion concentrations were determined by ICP-OES. Removal efficiency was calculated based on initial and equilibrium metal concentrations. The experiments were performed in triplicate, and the average values were reported This procedure was used to determine the capability of GO-ATP to selectively remove light and heavy rare earth elements (REEs) in the presence of competing cations such as Na^+^, K^+^, Ca^2+^, Mg^2+^, and Fe^3+^. The composition of the raffinate before and after treatment is presented in Table 7.

## 3. Results and discussion

### 3.1 Characterization of GO-ATP nanocomposite

#### 3.1.1. Fourier transform infrared (FTIR) spectroscopy

**Fig. 2:**
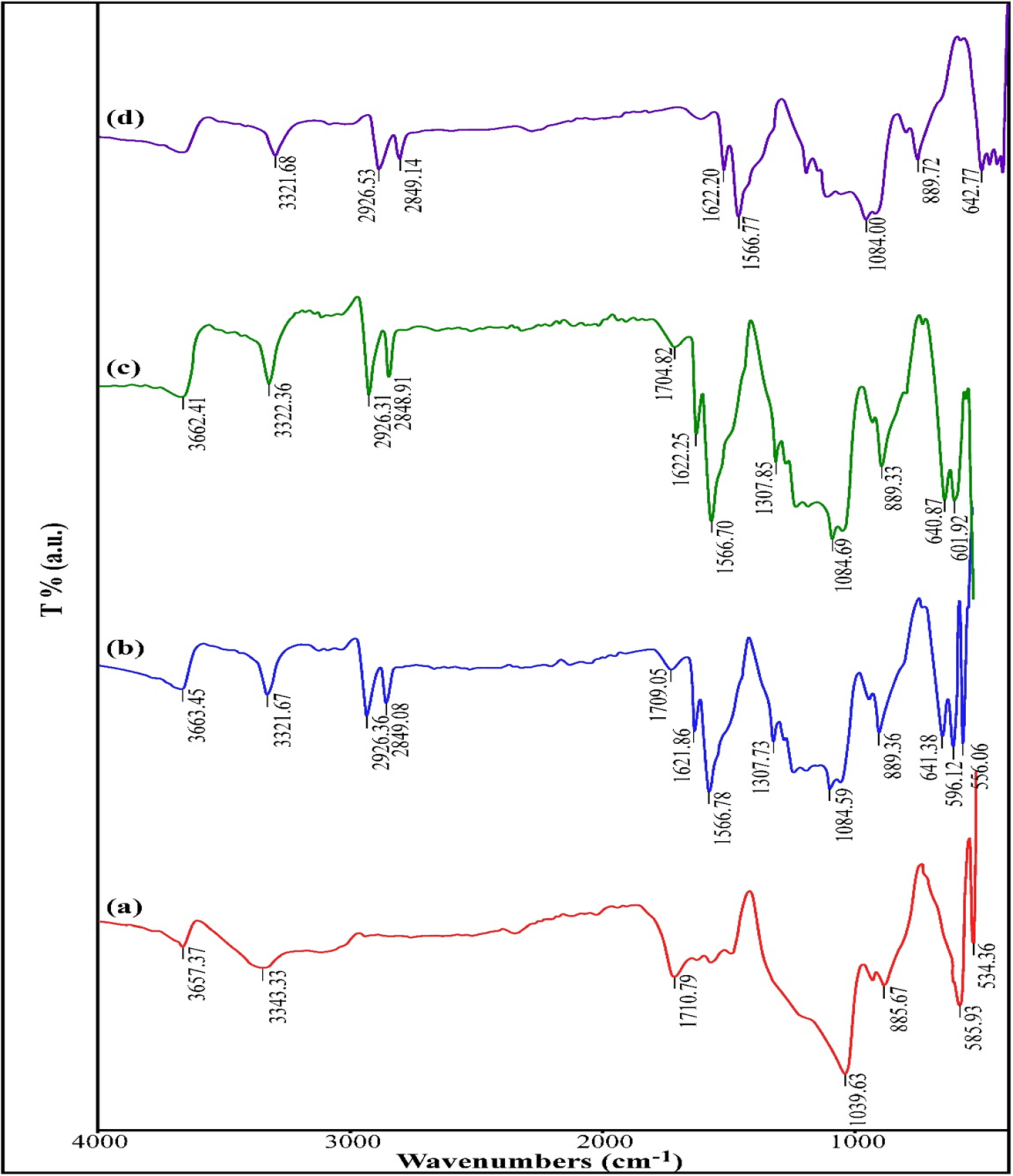
FTIR spectrum of GO (a), GO-ATP (b), GO-ATP loaded Nd^3+^ (c) and GO-ATP loaded Y^3+^ (d).

FTIR spectroscopy was employed to elucidate the functional groups involved in the adsorption process of Nd^3+^ and Y^3+^ onto the GO-ATP nanocomposite Fig. 2. The spectrum of pristine GO Fig. 2(a) displays broad bands around 3343 and 3657 cm^−1^, which are attributed to O–H stretching vibrations of hydroxyl groups and adsorbed water molecules.^22,23^ A prominent peak at 1710 cm^−1^ corresponds to C=O stretching from carboxylic acid groups, while bands at 1093 and 885 cm^−1^ are assigned to C–O and epoxy groups, respectively.^22^ Upon ATP functionalization Fig. 2(b), noticeable changes in the FTIR profile confirm successful modification of GO. New bands appear near 1621 cm^−1^ and 1307 cm^−1^, corresponding to the asymmetric stretching of phosphate (P=O) and P–O–C linkages, respectively, indicating the presence of ATP phosphate groups.^16^ Additionally, enhanced peaks at 2926 and 2849 cm^−1^ suggest C–H stretching from ATP ribose moiety. These spectral changes, together with the observed variation in surface charge from zeta potential analysis, demonstrate that the functionalization of GO with ATP occurs *via* chemical grafting rather than simple physical adsorption, confirming the formation of a chemically bonded GO-ATP nanocomposite.

Following Nd^3+^Fig. 2(c) and Y^3+^Fig. 2(d) adsorption, the FTIR spectra exhibit significant shifts and intensity changes in key functional regions. The peak at 1622 cm^−1^ shifts and becomes more intense, suggesting interaction between REE ions and the phosphate or carboxyl groups *via* inner-sphere complexation.^16,24^ Furthermore, reductions in the intensity of the O–H and P–O bands (*e.g.*, at 3321–3322 and 1084–1104 cm^−1^) imply that these groups are actively involved in metal ion binding. The slight shift and reduced transmittance in the P–O–C and C–O bands further support complex formation between REEs and oxygen donor atoms from ATP moieties.^25^ These spectral modifications confirm that phosphate, carboxyl, and hydroxyl groups are the primary binding sites responsible for Nd^3+^ and Y^3+^ adsorption onto the GO-ATP surface.

#### 3.1.2. Surface morphology and elemental composition analysis

**Fig. 3:**
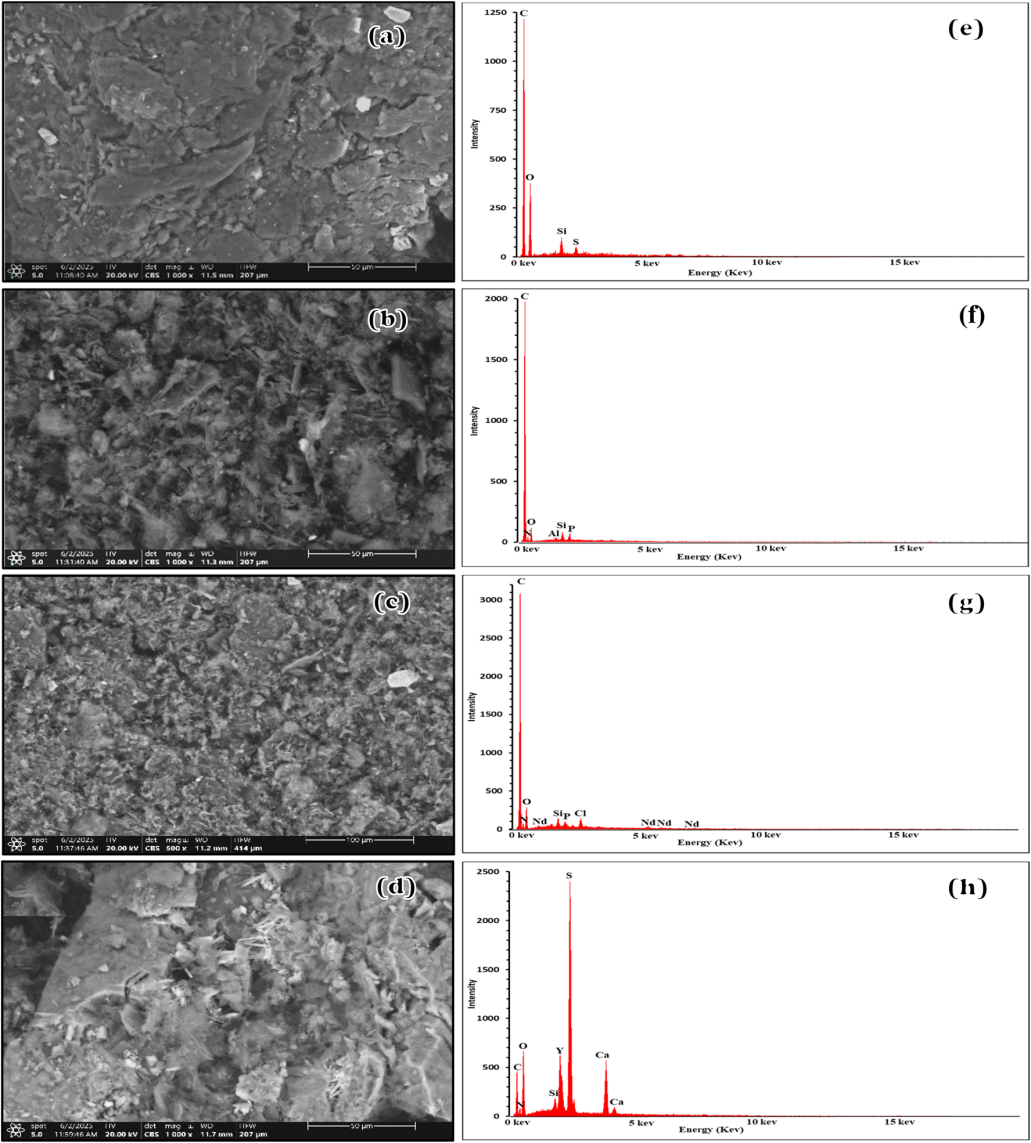
SEM micrographs (a–d) and EDX spectra (e–h) of graphene oxide, GO-ATP, GO-ATP loaded Nd^3+^ and GO-ATP loaded Y^3+^ respectively.

The surface morphologies and elemental compositions of the synthesized GO-ATP nanocomposite and its REE-loaded forms were characterized using scanning electron microscopy (SEM) and energy-dispersive X-ray spectroscopy (EDX), as presented in Fig. 3. SEM images Fig. 3(a–d) depict the surface structures of pristine graphene oxide (GO), GO-ATP, GO-ATP loaded with Nd^3+^, and GO-ATP loaded with Y^3+^, respectively, while corresponding EDX spectra Fig. 3(e–h) illustrate their elemental compositions. The SEM image of GO Fig. 3(a) displays ultrathin, wrinkled, and layered nanosheets, typical of exfoliated graphene oxide. This morphology contributes to the high surface area and exposes abundant oxygen-containing functional groups, enhancing its reactivity. After ATP modification Fig. 3(b), the surface becomes denser and rougher, indicative of successful anchoring of ATP molecules onto or between the GO layers. This structural transformation suggests the introduction of phosphate-rich moieties that can enhance adsorption affinity for REEs. Post-adsorption images reveal further morphological changes. The surface of GO-ATP after Nd^3+^ adsorption Fig. 3(c) shows increased agglomeration and roughness, likely due to coordination between Nd^3+^ ions and the phosphate and oxygenated functional groups present on the GO-ATP matrix. A similar behavior is observed in the Y^3+^-loaded composite Fig. 3(d), where the formation of aggregated clusters indicates successful ion binding.

EDX analysis corroborates these findings. The spectrum of GO Fig. 3(e) shows dominant peaks for carbon (C) and oxygen (O), confirming the presence of oxygenated functionalities. In the GO-ATP composite Fig. 3(f), the emergence of phosphorus (P) peaks confirms the incorporation of ATP. For Nd^3+^ and Y^3+^ loaded composites Fig. 3(g) and (h), the presence of Nd and Y peaks, respectively, affirms the successful adsorption of the target REEs. Notably, phosphorus peaks remain visible post-adsorption, underscoring the continued presence of ATP as a key binding component. Together, the SEM and EDX results confirm that ATP modification effectively enhances the structural and chemical properties of GO, enabling the composite to serve as an efficient and selective adsorbent for rare earth ions such as Nd^3+^ and Y^3+^.

#### 3.1.3. Transmission electron microscopy (TEM) analysis

**Fig. 4:**
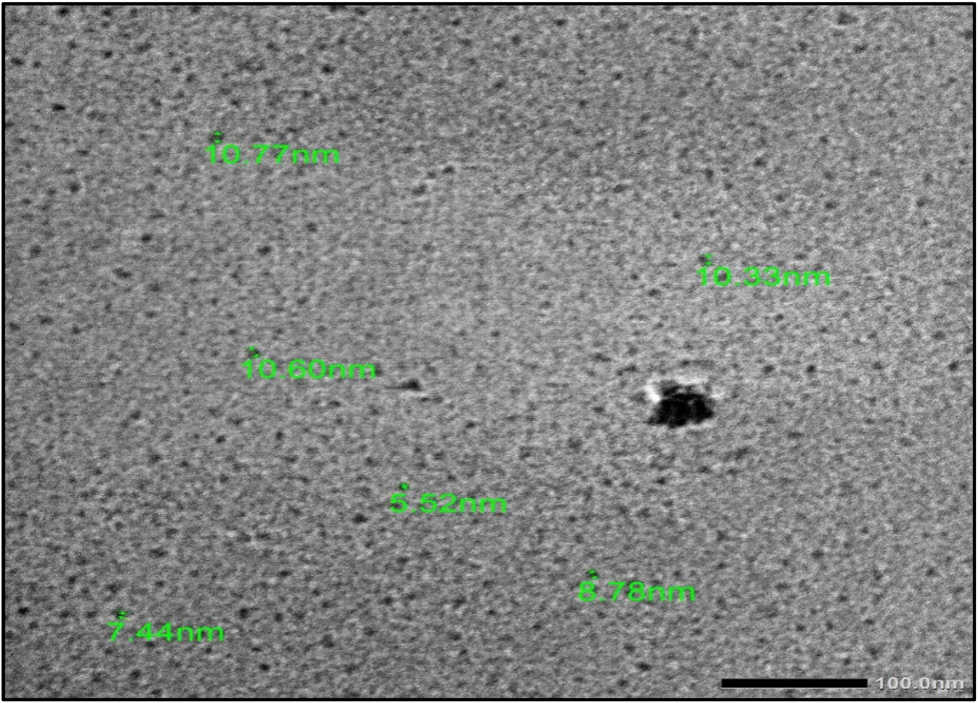
TEM micrograph of GO-ATP adsorbent.

As shown in Fig. 4, the sample exhibits well-defined, rounded mesopores with diameters ranging between 5.32 and 10.8 nm, consistent with the pore size distribution obtained from BJH and NLDFT analyses. The observed pore diameters align with mesoporous characteristics, confirming the material's hierarchical porosity. The pore structure appears relatively uniform and dispersed, indicating efficient exfoliation of graphene oxide sheets and intercalation of ATP molecules. Some denser regions may correspond to ATP aggregates or partially restacked GO layers, which contribute to the observed ink-bottle-type pores confirmed by the desorption branch of the BJH analysis (40.07 nm). The nanoscale architecture supports the material's potential for rapid diffusion and high surface accessibility during adsorption processes.

### 3.2 Surface area and porosity analysis

**Fig. 5:**
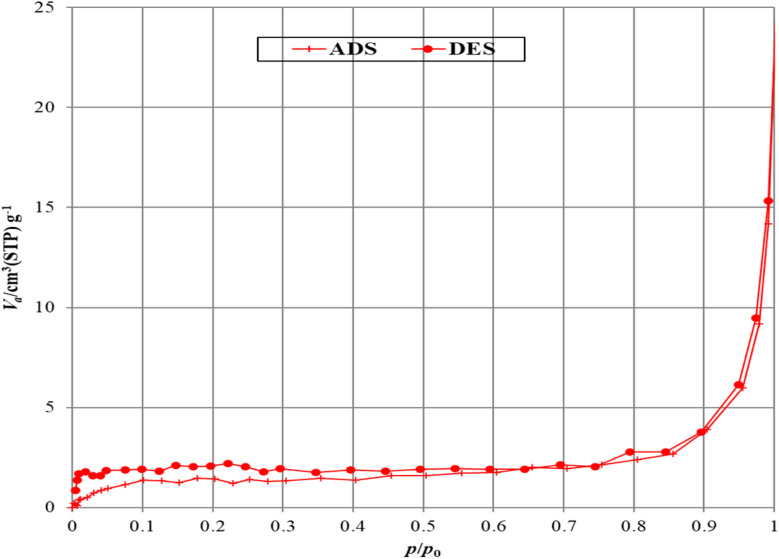
Nitrogen adsorption–desorption isotherm of GO-ATP measured at 77 K. The curve shows a Type IV isotherm with an H3-type hysteresis loop, confirming the mesoporous structure with slit-like pores.

The textural characteristics of the GO-ATP nanocomposite were evaluated using nitrogen adsorption–desorption isotherms at 77 K. The adsorption–desorption isotherm presented in Fig. 5 displays a Type IV pattern accompanied by an H3 hysteresis loop, signifying the presence of a mesoporous material characterized by slit-shaped or plate-like pores. A pronounced uptake at high relative pressures (*p*/*p*° > 0.9) is consistent with capillary condensation within mesopores, whereas the absence of a steep rise at low relative pressures (*p*/*p*° < 0.1) suggests negligible microporosity. The BET surface area was calculated to be 5.64 m^2^ g^−1^, significantly reduced compared to unmodified graphene oxide, confirming the partial blockage of surface area due to ATP grafting. This reduction was further supported by Langmuir analysis, which yielded a surface area of 1.32 m^2^ g^−1^, indicative of limited monolayer coverage and heterogeneous surface behavior. The total pore volume at *p*/*p*° = 0.990 was 0.0208 cm^3^ g^−1^, and the average pore diameter was 14.80 nm, indicating mesoporosity. BJH analysis revealed pore size peaks at 1.50 nm (adsorption branch) and 40.07 nm (desorption branch), highlighting the presence of ink-bottle-type mesopores. The t-Plot and DA models confirmed minimal microporosity, while NLDFT/GCMC analysis identified a hierarchical pore structure with peaks at 1.91 nm (area based) and 44.24 nm (volume based), and a total pore volume of 0.0246 cm^3^ g^−1^. These results suggest that despite surface area reduction, the GO-ATP nanocomposite retains diverse mesoporous architecture suitable for adsorption applications. Notably, despite the significant reduction in BET surface area, the GO-ATP nanocomposite exhibits superior adsorption performance when normalized to surface area compared to pristine graphene oxide. This behavior indicates that adsorption is governed predominantly by specific chemical interactions rather than by physical surface area alone. The introduction of ATP moieties provides high-density phosphate and nitrogen donor sites, which enhance coordination interactions with trivalent rare earth ions, thereby compensating for the loss of accessible surface area (Table 1).

**Table 1:** Summary of key surface area and porosity parameters of the synthesized material

Method	Parameter	Value	Unit
BET	Surface area	5.64	m^2^ g^−1^
	Total pore volume (*p*/*p*_0_ = 0.990)	0.0208	cm^3^ g^−1^
	Average pore diameter	14.80	N m
Langmuir	Surface area	1.32	m^2^ g^−1^
BJH (adsorption)	Peak pore diameter	1.50	N m
	Average pore diameter	8.23	N m
	Pore volume	0.0272	cm^3^ g^−1^
BJH (desorption)	Average pore diameter	40.07	N m
	Pore volume	0.0222	cm^3^ g^−1^
t-Plot	Micropore volume	n.d.	cm^3^ g^−1^
DA	Micropore volume	0.00587	cm^3^ g^−1^
NLDFT/GCMC	Peak pore diameter (area-based)	1.91	N m
	Peak pore diameter (volume-based)	44.24	N m
	Pore volume	0.0246	cm^3^ g^−1^

#### 3.2.1. XRD analysis

**Fig. 6:**
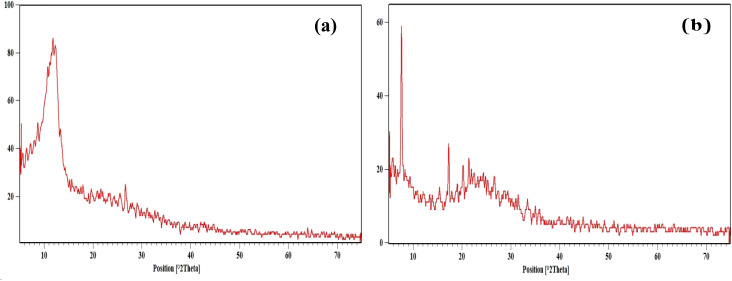
XRD for GO (a) and GO-ATP (b).

The crystalline structure and phase composition of the synthesized graphene oxide and its nanocomposite with adenosine triphosphate were examined using X-ray diffraction (XRD), as shown in Fig. 6. The XRD pattern of Fig. 6(a) exhibits a characteristic sharp peak centered at 2*θ* = 11°, which corresponds to the (001) reflection of graphene oxide. This peak signifies the successful oxidation of graphite (ICSD no. 171799) and the subsequent formation of oxygen-containing functional groups (*e.g.*, hydroxyl, epoxide, and carboxyl), which increase the interlayer spacing of graphene sheets due to the incorporation of water molecules and functional groups. The broadness of the peak reflects a disordered structure with small crystallite size. A weak hump between 20°–30° suggests traces of unexfoliated graphite or disordered carbon. In contrast graphene oxide-adenosine triphosphate nanocomposite Fig. 6(b) exhibits a sharp and intense peak at 2*θ* = 7.5°, indicating a further increase in interlayer spacing due to successful intercalation of ATP molecules. The high intensity and sharpness of this peak suggest improved structural order and crystallinity. Additional minor peaks between 18°–23° may arise from residual GO, ATP, or new composite phases (ICSD no. 181305 and 35032). Overall, the shift from a broad GO peak to a sharp low-angle reflection in Fig. 6(b) confirms the successful formation of the GO-ATP nanocomposite with expanded interlayer spacing and enhanced structural ordering.

#### 3.2.2. Zeta potential and surface charge analysis

**Fig. 7:**
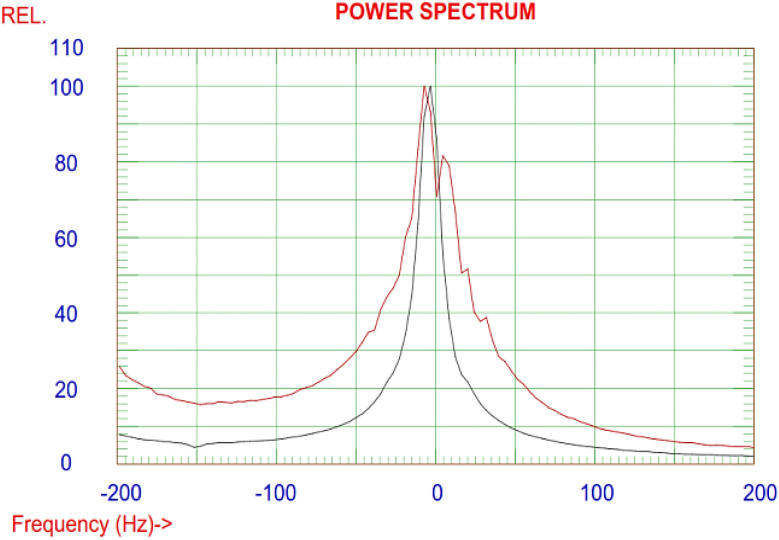
Zeta potential of the GO-ATP using NICOMP 380 ZLS (He–Ne laser, 632 nm, 18.9°); samples dispersed in Millipore water (0.2 g/5 mL) and ultrasonicated for 5 min.

The surface charge behavior of the synthesized GO-ATP composite was investigated by zeta potential measurements to assess its colloidal stability and adsorption capability in aqueous media. As presented in Fig. 7, GO-ATP exhibited a pronounced negative zeta potential of −47.31 mV, indicating strong electrostatic stabilization and excellent dispersion in water. Such high negative surface potential is attributed to the abundance of deprotonated oxygen-containing functional groups on graphene oxide (–COO^−^, –OH) together with phosphate groups (–PO_4_^3−^) introduced through ATP modification. The sharp peak observed in the corresponding power spectrum further confirms the formation of a homogeneous and stable suspension with minimal agglomeration tendencies. The strongly negative surface charge enhances the electron-donating ability of GO-ATP and promotes electrostatic attraction and surface complexation with trivalent rare earth cations such as Nd^3+^ and Y^3+^ during adsorption. These findings are consistent with HSAB principles, where hard Lewis base groups (O-donor atoms) preferentially coordinate with hard Lewis acid REE ions. Therefore, the significant negative surface potential of GO-ATP not only ensures colloidal stability but also provides a favorable driving force for REE uptake, supporting its potential application in hydrometallurgical wastewater treatment and selective REE recovery from acidic effluents.

### 3.3 Adsorption of ions on GO-ATP

#### 3.3.1. Effect of pH on Nd^3+^ and Y^3+^ adsorption

**Fig. 8:**
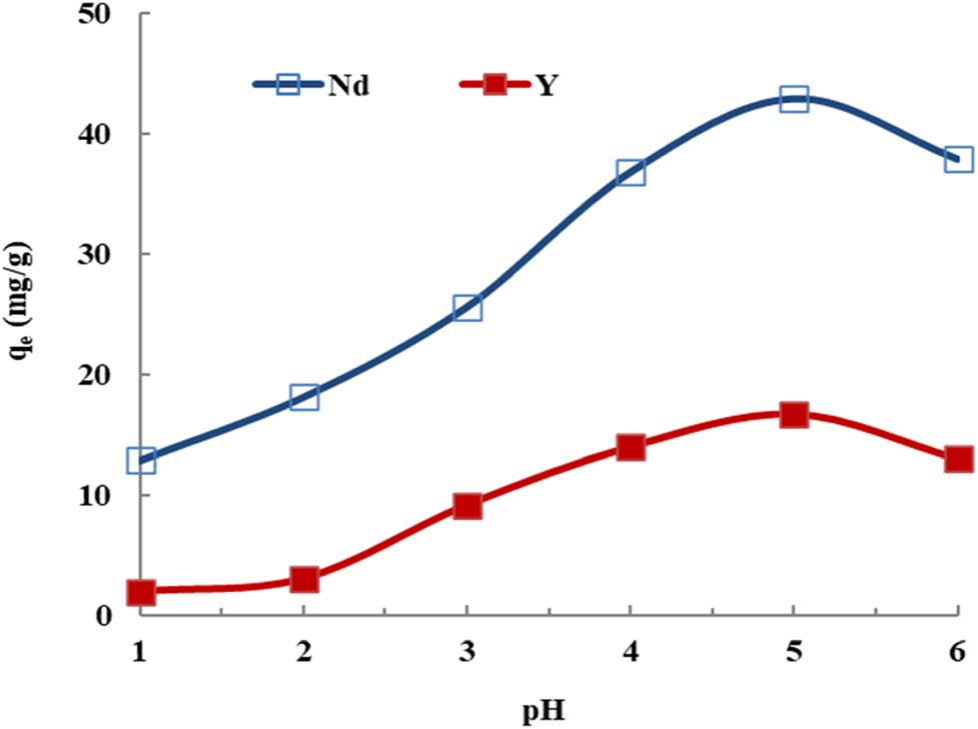
Effect of pH on Nd and Y adsorption using GO-ATP at 230 rpm, 25 °C, 60 min, and 50 ppm.

The effect of solution pH on the adsorption capacity of Nd^3+^ and Y^3+^ ions onto the GO-ATP nanocomposite is illustrated in Fig. 8. A gradual increase in adsorption capacity was observed with increasing pH, reaching a maximum at pH 5 for both ions. Nd^3+^ exhibited a significantly higher adsorption capacity (44 mg g^−1^) compared to Y^3+^ (17 mg g^−1^), indicating a stronger affinity toward the functional groups of the composite. This behavior can be attributed to enhanced electrostatic attraction and surface complexation between the trivalent metal ions and the deprotonated functional groups, including carboxyl, hydroxyl, and phosphate moieties present within the GO-ATP structure.^21^ The optimum pH of 5 was selected because it maximized adsorption efficiency while minimizing significant REE hydrolysis or precipitation under the investigated experimental conditions. In practical applications, such pH conditions can be maintained using controlled acid/base addition or appropriate buffering strategies commonly employed in hydrometallurgical operations. At lower pH values (pH < 3), protonation of surface active sites and competitive binding of H^+^ ions significantly hinder metal uptake.^21,26^ As the pH increases, deprotonation of the active groups enhances the negative surface charge, favoring the adsorption of positively charged rare earth ions. Beyond pH 5, a slight decline in adsorption is likely due to partial precipitation of metal hydroxides or saturation of binding sites, consistent with previous observations in phosphate-functionalized and GO-based adsorbents.^27^ The separation of chemically similar REEs remains a major challenge due to their comparable trivalent oxidation states and coordination chemistry. Nevertheless, the GO-ATP adsorbent demonstrated preferential interaction tendencies that may be associated with differences in ionic radius, hydration energy, and coordination affinity toward phosphate and oxygen containing functional groups. The stronger interaction of smaller and more highly hydrated ions may contribute to partial selectivity during adsorption/desorption processes. These results prove that pH is a critical factor influencing both the adsorption capacity and selectivity of the GO-ATP nanocomposite.

#### 3.3.2. Effect of contact time on adsorption kinetics

**Fig. 9:**
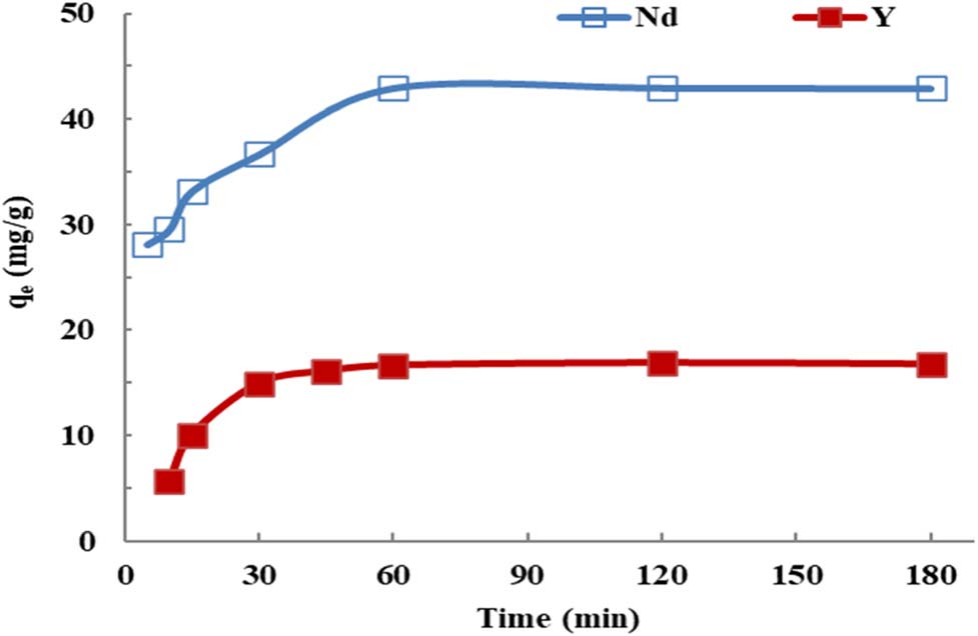
Effect of contact time on Nd and Y adsorption using GO-ATP.

The adsorption kinetics of Nd^3+^ and Y^3+^ onto the GO-ATP nanocomposite revealed a rapid initial uptake phase, with significant removal efficiencies observed within the first 10–20 minutes of contact, Fig. 9. This fast adsorption is attributed to the abundance of active binding sites on the surface of the nanocomposite, facilitating electrostatic attraction and surface complexation with the rare earth ions. As the contact time progressed, the adsorption rate decreased, reaching equilibrium near 60 minutes. This plateau suggests progressive saturation of available binding sites and reduced ion mobility, characteristic of systems governed by pseudo-second-order kinetics and intraparticle diffusion mechanisms. Such kinetic behavior is typically associated with chemisorption processes, involving valence forces through electron sharing or exchange between sorbent and sorbate.^21,28^ Although equilibrium was attained after approximately 60 min, this adsorption rate remains within the typical range reported for functionalized nanoadsorbents operating through coordination-driven adsorption mechanisms. The relatively moderate adsorption kinetics may be attributed to the combined effects of surface complexation reactions and intraparticle diffusion within the GO-ATP structure. In these systems, adsorption proceeds through coordination interactions between REE ions and phosphate and oxygen containing functional groups rather than weak physical adsorption alone. Consequently, the adsorption process may occur more slowly than purely physisorption-controlled systems, which often exhibit faster but less selective uptake behavior. Therefore, the observed equilibrium time reflects a balance between adsorption rate, binding strength, and selective interaction toward REE ions. This behavior further supports the proposed inner-sphere complexation mechanism between REE ions and ATP functional groups.

#### 3.3.3. Kinetic models for Nd^3+^ and Y^3+^ adsorption onto GO-ATP

The adsorption kinetics of Nd^3+^ and Y^3+^ onto graphene oxide-adenosine triphosphate (GO-ATP) nanocomposites were investigated using multiple kinetic models to elucidate the rate-controlling mechanisms and adsorption behavior. These models are widely applied in studies involving REEs adsorption onto functionalized nanomaterials.

##### 3.3.3.1. Pseudo-first-order model

**Fig. 10:**
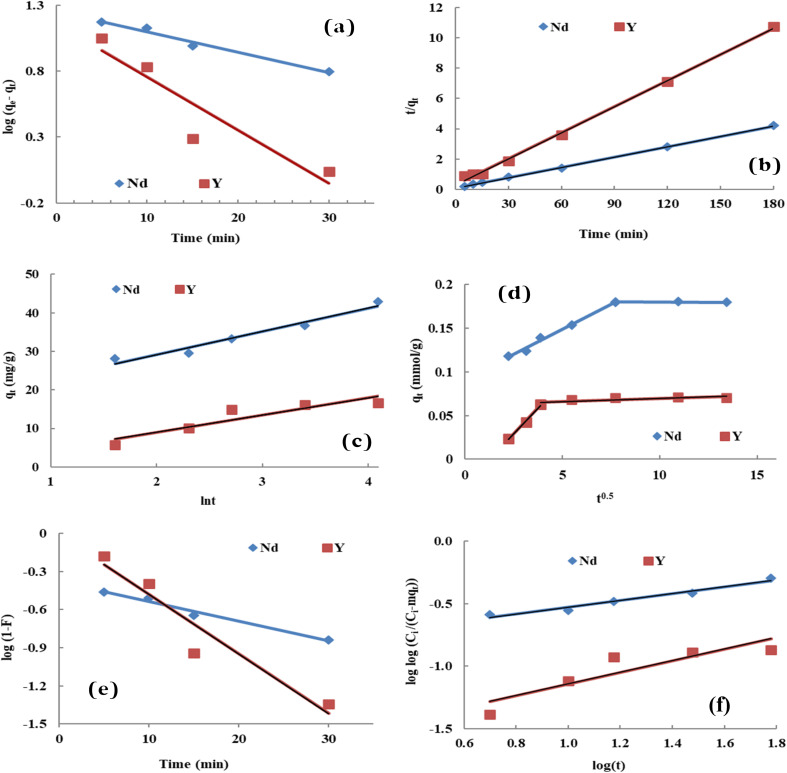
Kinetic data for the adsorption of Nd and Y ions by GO-ATP adsorbents: Lagergren pseudo-first order (a), pseudo-second order (b), Elovich (c), intraparticle diffusion (d), film diffusion (e), and Bangham (f) models.

The pseudo-first-order model describes the initial adsorption stage, assuming that the rate of uptake is proportional to the difference between equilibrium adsorption capacity and the amount adsorbed at time *t*. It is primarily applicable to systems where physisorption or surface diffusion dominates. The linear form of the model is given by:4
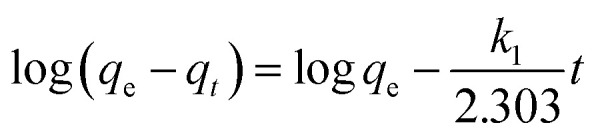
where; *q*_e_ and *q*_*t*_ are the amounts of the adsorbed Nd^3+^ and Y^3+^ ions (mg g^−1^) at the equilibrium time and at any instant of time “*t*” respectively, whereas *k*_1_ is the Lagergren constant (l/min). This model moderately fits the data, especially for Nd^3+^ (*R*^2^ = 0.978). The noticeable difference between the calculated *q*_e_ values (17.82 mg g^−1^ for Nd^3+^ and 14.31 mg g^−1^ for Y^3+^) and those obtained experimentally (Fig. 10(a) and Table 2) implies that adsorption on GO-ATP does not follow pseudo-first-order kinetics alone, suggesting the involvement of additional rate-controlling mechanisms.^29,30^

**Table 2:** Kinetic data for the adsorption of Nd and Y ions by GO-ATP adsorbents

Kinetic model	Model parameters	Nd	Y
Experimental	*q*_e_(mg g^−1^)	42.93	16.88
Pseudo-first order kinetics	*q*_1st_, (mg g^−1^)	17.8197	14.312
	*k*_1_, (min^−1^)	0.03547	0.0926
	*R* ^2^	0.978	0.8517
Pseudo-second order kinetics	*q*_2nd_, (mg g^−1^)	44.0529	17.452
	*k*_2_, (g mg^−1^ min^−1^)	0.00553	0.1084
	*R* ^2^	0.9995	0.9978
Elovich kinetic model	*β* (g mg^−1^)	0.16557	0.22281
	*α* (mg g^−1^ min^−1^)	0.22281	4.49761
	*R* ^2^	0.9583	0.842
Intraparticle diffusion model	*k*_ip_, (mg g^−1^ min^−0.5^)	0.0115	0.0236
	*I*	0.0.0917	0.0301
	*R* ^2^	0.9898	0.9886
Liquid film diffusion model	*k*_fd_	0.035466	0.108011
	*R* ^2^	0.978	0.9178
Bangham kinetic model	*k*_b_ (mL g^−1^ L^−1^)	18.2542	2.84422
	*A*	0.2711	0.4646
	*R* ^2^	0.9628	0.8796

##### 3.3.3.2. Pseudo-second-order model (Ho & McKay)

This model assumes that chemisorption is the rate-limiting step, involving electron exchange or valence forces between adsorbent and adsorbate. The linear form is expressed as:5
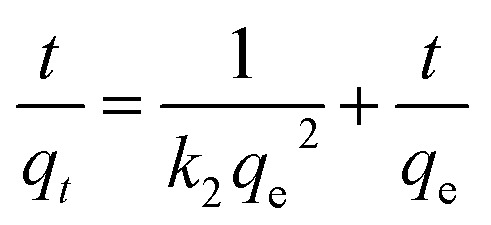
where *q*_*t*_ and *q*_e_ are adsorption capacities at time *t* and equilibrium, respectively, and *k*_2_ is the rate constant. This model fits exceptionally well for both ions, with very high correlation coefficients (*R*^2^ = 0.9995 for Nd^3+^ and 0.9978 for Y^3+^). The predicted *q*_e_ values (44.05 mg g^−1^ for Nd^3+^ and 17.45 mg g^−1^ for Y^3+^) closely match the experimental values, further confirming the applicability of this model, Fig. 10(b) and Table 2.^31,32^ These results indicate that the adsorption process is predominantly governed by chemisorption, involving inner-sphere complexation between REE ions and phosphate/nitrogen donor sites of ATP. This coordination-driven mechanism is consistent with the functional groups identified by FTIR analysis and supported by thermodynamic findings.

##### 3.3.3.3. Elovich model

The Elovich equation accounts for chemisorption on heterogeneous surfaces, often characterized by diverse activation energies:6
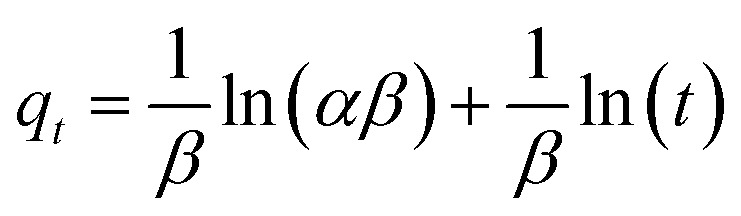
where *α* (mg g^−1^ min^−1^) is the initial adsorption rate, and *β* (g mg^−1^) is the desorption constant. It provided a reasonable fit for Nd^3+^ (*R*^2^ = 0.9583) but less so for Y^3+^ (*R*^2^ = 0.842), suggesting that the GO-ATP surface has more diverse energy barriers for Nd^3+^ adsorption, Fig. 10(c). The higher initial rate constant (*α*) for Y^3+^ (4.50 mg g^−1^ min^−1^) may reflect faster initial uptake, although overall adsorption is lower.^11,33^

##### 3.3.3.4. Intraparticle diffusion model

This model examines whether the adsorption process is controlled by the diffusion of metal ions into the internal pores of the GO-ATP structure. It is defined as:

7 *q*_*t*_ = *k*_ip_*t*^0.5^ + *I*

The intraparticle diffusion plots yielded high *R*^2^ values (0.9898 for Nd^3+^, 0.9886 for Y^3+^), indicating that pore diffusion contributes to the rate-limiting step. However, the non-zero intercepts (*I* = 0.0917 and 0.0301) suggest that intraparticle diffusion is not the sole controlling mechanism and is preceded by boundary layer diffusion, Fig. 10(d).^34,35^

##### 3.3.3.5. Liquid film diffusion model

This model evaluates whether mass transport through the boundary layer surrounding the adsorbent limits the adsorption rate. It is given as:8
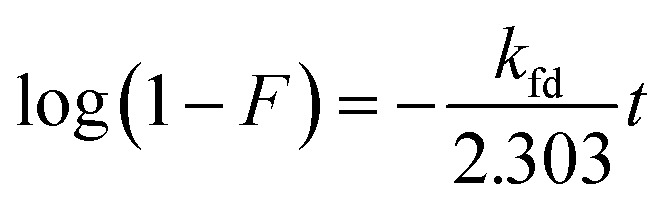
where *F* = *q*_*t*_/*q*_e_ is the fractional attainment of equilibrium, and *k*_fd_ is the film diffusion rate constant (min^−1^). Fig. 10(e) and Table 2 illustrated that, *R*^2^ values were moderately high (0.978 for Nd^3+^ and 0.918 for Y^3+^), indicating some influence of boundary layer resistance. However, the model's lower predictive accuracy and non-zero intercepts confirm that film diffusion is not the dominant mechanism.^36^

##### 3.3.3.6. Bangham model

The Bangham model can be applied to adsorption systems where the rate-determining step is intraparticle diffusion. Eqn (9) was used to apply the adsorption data to the Bangham model.^37^9
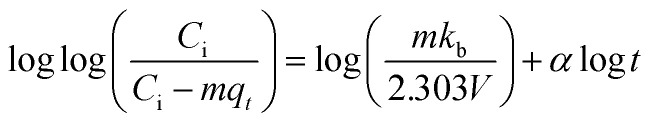
where *V* is the volume of solution (mL), *m* is the weight of adsorbent per liter of solution (g L^−1^), *α* and *k*_b_ (mL g^−1^ L^−1^) are Bangham constants. Plots of the adsorption time data using the Bangham model are displayed in Fig. 10(f). This model reveals pore diffusion under concentration gradients.

It fits better for Nd^3+^ (*R*^2^ = 0.9628) than for Y^3+^ (*R*^2^ = 0.8796), supporting the notion that Nd^3+^ has stronger pore level interactions with GO-ATP. The higher Bangham constant (*k*_b_ = 18.25) for Nd^3+^ further reflects its greater accessibility to the internal surface. Based on the kinetic results presented in Fig. 10 and Table 2, the adsorption behavior of Nd^3+^ and Y^3+^ ions onto GO-ATP was best described by the pseudo-second-order (PSO) model, as evidenced by the high correlation coefficients (*R*^2^ = 0.9995 for Nd^3+^ and 0.9978 for Y^3+^) and the close agreement between calculated and experimental adsorption capacities. This indicates that the adsorption process is predominantly governed by chemisorption, involving strong interactions between the rare earth ions and the phosphate and oxygenated functional groups present on the surface of GO-ATP. To further elucidate the adsorption mechanism, diffusion-based kinetic models were applied. The intraparticle diffusion model showed relatively high correlation coefficients (*R*^2^ = 0.9898 for Nd^3+^ and 0.9886 for Y^3+^), suggesting that pore diffusion contributes to the overall rate of adsorption. However, the non-zero intercepts of the plots indicate that intraparticle diffusion is not the sole rate-controlling step, and boundary layer effects are also involved. This was further supported by the liquid film diffusion model, which demonstrated good fitting (*R*^2^ = 0.978 for Nd^3+^ and 0.9178 for Y^3+^), implying that film diffusion controls the initial stage of adsorption, especially in the case of Y^3+^, which exhibited a higher film diffusion coefficient (*k*_fd_ = 0.1080). The applicability of the Elovich model confirms the contribution of chemisorption on a heterogeneous surface, while the Bangham model supports the involvement of pore diffusion in the adsorption process due to the mesoporous structure of GO-ATP. Overall, the kinetic evaluation reveals that the adsorption of Nd^3+^ and Y^3+^onto GO-ATP proceeds *via* a complex multi-step mechanism, primarily driven by chemisorption with secondary contributions from both film and intraparticle diffusion.

#### 3.3.4. Effect of initial concentration and temperature on Nd^3+^ and Y^3+^ adsorption

**Fig. 11:**
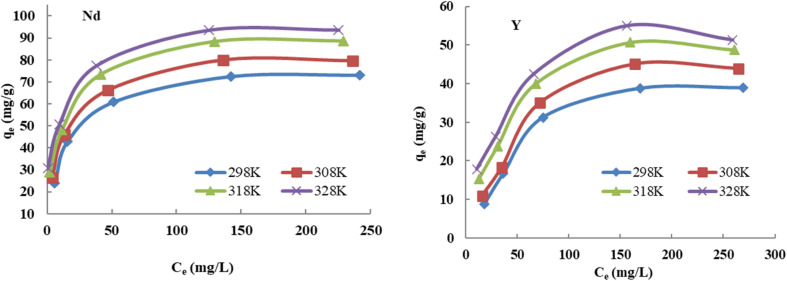
Effect of initial concentration on adsorption of Nd and Y using GO-ATP adsorbents at different temperatures.

The adsorption behavior of Nd^3+^ and Y^3+^ ions onto the GO-ATP nanocomposite was strongly influenced by both initial ion concentration and solution temperature, as demonstrated in Fig. 11. These parameters critically affect the adsorbent's efficiency by altering site accessibility, adsorption capacity, and interaction thermodynamics. At lower initial ion concentrations (20–50 mg L^−1^), the removal efficiencies for both Nd^3+^ and Y^3+^ were remarkably high approaching nearly 100%. This behaviour is attributed to the abundance of accessible active sites on the GO-ATP surface, which readily bind the metal ions. However, as the concentration increased to 300 mg L^−1^, a notable decline in removal efficiency was observed. This is indicative of site saturation, where the fixed number of adsorption sites on the nanocomposite surface becomes insufficient to accommodate the higher ionic load. Notably, Nd^3+^ consistently exhibited a higher removal efficiency than Y^3+^ across the concentration range, which is consistent with its higher experimental adsorption capacity and likely stronger electrostatic and coordination interactions with phosphate and oxygen-containing functional groups.^38^ Simultaneously, temperature had a synergistic effect on the adsorption performance. With an increase in temperature from 20 °C to 55 °C, the removal efficiencies of both ions improved significantly. This temperature-dependent enhancement suggests that the adsorption process is endothermic, where thermal energy facilitates greater interaction between the adsorbate and the adsorbent. The increase in temperature likely enhances ion mobility, overcomes activation energy barriers, and promotes stronger interactions between the metal ions and functional groups on GO-ATP. The effect was more pronounced for Nd^3+^, reaffirming its stronger affinity for the GO-ATP surface.^38^

#### 3.3.5. Adsorption isotherms of Nd^3+^ and Y^3+^onto GO-ATP nanocomposite

The equilibrium adsorption behavior of Nd^3+^ and Y^3+^ onto GO-ATP was analyzed using Langmuir, Freundlich, Dubinin–Radushkevich (D–R), and Temkin isotherm models as illustrated in Table 3, at various temperatures (298–328 K), as summarized in Table 4. These models provide insight into the adsorption mechanism, surface properties, and energetic interactions between the adsorbate ions and the adsorbent surface.^39–42^

**Table 3:** Summary of adsorption isotherm models and thermodynamics

Isotherms model	Equation	Parameters	Reference
Langmuir	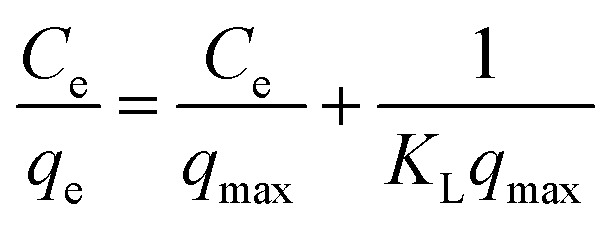 (10)	*q*_max_: max capacity; *b*: Langmuir constant	39
	Separation factor constant (*R*_L_)	*R*_L_: avorable adsorption	
	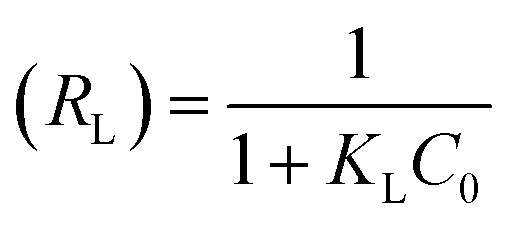 (11)		
Freundlich	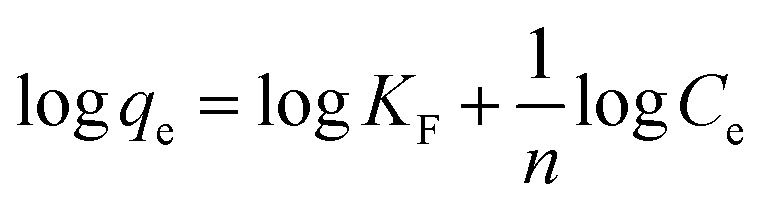 (12)	*K*_F_, 1/*n*: Freundlich constants	40
Dubinin–Radushkevich	Ln *q*_e_ = ln *Q*_D_ − *βε*^2^ (13)	*ε* = *RT* ln(1 + 1/*C*_e_); *β*: energy constant	41
	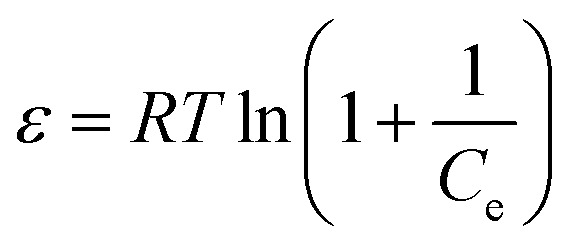 (14)	*E*: mean free energy	
	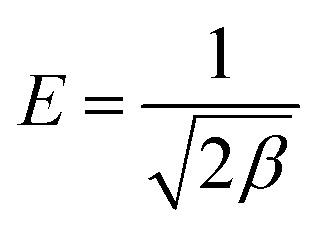 (15)		
Temkin	*q*_e_ = *β* ln *A*_T_ + *β* ln *C*_e_ (16)	*B* = *RT*/*bT*; *A*: Temkin constants	42
	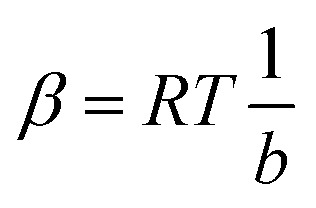 (17)		
Gibbs free energy	Δ*G*° = −*RT* ln *k*_L_ (18)	*R*: gas constant; *T*: temperature; *K*_c_: distribution constant	43
	Δ*G*° = Δ*H*° − *T*Δ*S*° (19)		
Van't Hoff equation	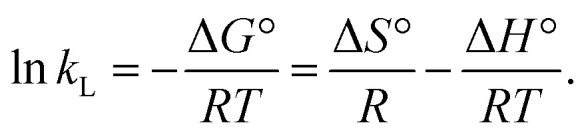 (20)	Δ*H*°, Δ*S*°: enthalpy and entropy changes	43

**Table 4:** Adsorption isotherms of Nd and Y ions by GO-ATP adsorbents

Isotherms model	Parameters	Nd	Y	Temperature
Langmuir isotherm	*q*_max,_ (mg g^−1^)	77.5194	50.7614	298K
		89.2857	55.8659	308 K
		91.7431	56.8182	318 K
		96.1538	58.1395	328 K
	*K*_L,_ (L mg^−1^)	0.0782	0.0145	298K
		0.0948	0.0171	308 K
		0.1290	0.0297	318 K
		1.7247	0.0392	328 K
	*R* ^2^	0.9997	0.9715	298 K
Freundlich isotherm	*K*_F,_ (mg g^−1^)	16.6802	2.10426	298 K
	*n*	3.40948	1.79083	
	*R* ^2^	0.9077	0.9002	
D–R isotherm	*q*_max_, (mg g^−1^)	70.1124	32.3657	298 K
	*β*, (mol^2^ kJ^−2^)	2.0 × 10^−8^	7.0 × 10^−8^	
	*E*, (kJ mol^−1^)	5	2.673	
	*R* ^2^	0.9346	0.8907	
Temkin isotherm	*β* (kJ mol^−1^)	16.453	14.218	298 K
	*A*_T_ (L g^−1^)	1.24639	0.8214	
	*b*	150.58	174.26	
	*R* ^2^	0.9838	0.9833	

**Fig. 12:**
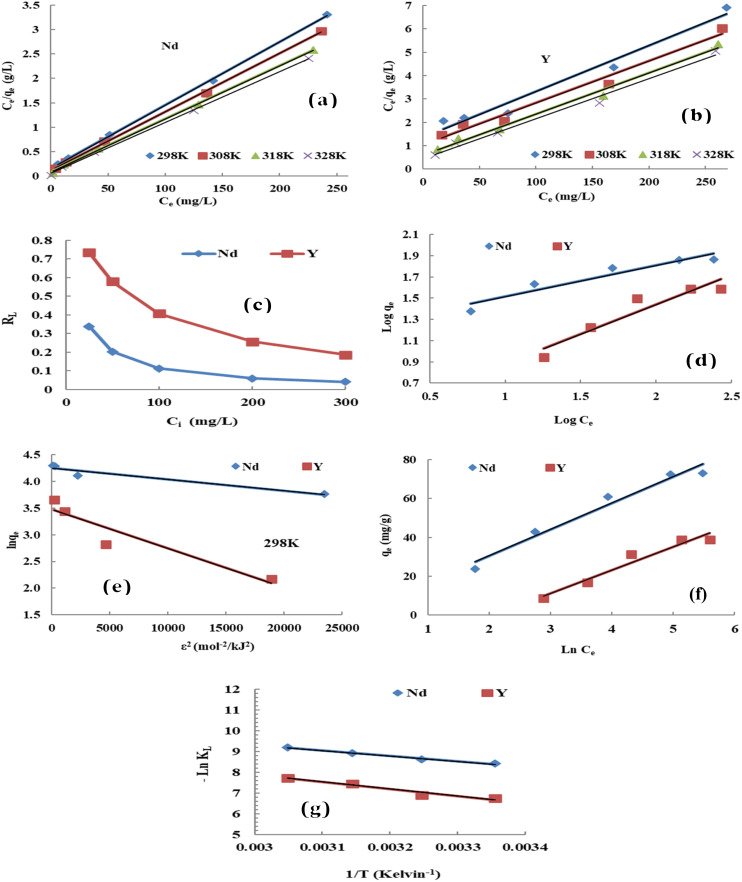
Isotherms and thermodynamic plots for the adsorption of Nd and Y ions by GO-ATP adsorbents: Langmuir (a and b), separation factors (c), Freundlich (d), Dubinin–Radushkevich (e), Temkin (f), and Van't Hoff plots (g).

The Langmuir isotherm yielded the best fit for both Nd^3+^ and Y^3+^ ions, with an *R*^2^ value of 0.9997 for Nd^3+^ and 0.9715 for Y^3+^, indicating monolayer adsorption onto a homogenous surface. The maximum adsorption capacity (*q*_max_) for Nd^3+^ increased from 77.52 to 96.15 mg g^−1^ with rising temperature, reflecting the endothermic nature of the adsorption process. Similarly, *q*_max_ for Y^3+^ increased from 50.76 to 58.14 mg g^−1^ over the same temperature range, Fig. 12(a and b). The Langmuir affinity constant (*K*_L_) increased significantly for Nd^3+^ (0.0782 to 1.7247 L mg^−1^), indicating enhanced binding strength at elevated temperatures. These findings suggest that GO-ATP exhibits a stronger adsorption affinity and higher capacity for Nd^3+^ than Y^3+^, Fig. 12(c). The Freundlich model, which accounts for surface heterogeneity and multilayer adsorption, also described the system adequately, with *R*^2^ values of 0.9077 and 0.9002 for Nd^3+^ and Y^3+^, respectively. The Freundlich constant (*K*_F_) for Nd^3+^ was 16.68 mg g^−1^ compared to 2.10 mg g^−1^ for Y^3+^, and the *n* values exceeded 1 in both cases, indicating favorable adsorption, Fig. 12(d). However, the lower correlation coefficients suggest that the Langmuir model better represents the equilibrium behavior for both ions. The D–R model exhibited *R*^2^ values of 0.9346 for Nd^3+^ and 0.8907 for Y^3+^, lower than the Langmuir model but sufficient to infer the adsorption nature. The calculated mean adsorption energy (*E*) values were 5 kJ mol^−1^ for Nd^3+^ and 2.67 kJ mol^−1^ for Y^3+^, indicating that the adsorption process is predominantly physical, involving weak electrostatic interactions rather than ion exchange, Fig. 12(e).^41^ The Temkin isotherm, which considers adsorbate–adsorbent interactions and a linear decrease in the heat of adsorption, showed *R*^2^ values of 0.9838 for Nd^3+^ and 0.9833 for Y^3+^. The heat of adsorption constants (*β*) were 16.45 and 14.22 kJ mol^−1^ for Nd^3+^ and Y^3+^, respectively, suggesting moderate interaction energies in both cases. The Temkin binding constants (*A*_T_) also supported the stronger affinity of GO-ATP for Nd^3+^, Fig. 12(f). These results demonstrate that the superior fitting of the Langmuir isotherm model confirms that the adsorption of Nd^3+^ and Y^3+^ onto GO-ATP occurs predominantly as monolayer coverage on a homogeneous surface, where each adsorption site can accommodate only one metal ion. The adsorption process is governed by specific chemisorption interactions, primarily through coordination with phosphate groups (–PO_4_^3−^) introduced by ATP and hydroxyl groups (–OH) on the GO framework, which serve as the main active binding sites for rare earth ions. The higher Langmuir adsorption capacity (*q*_max_) and affinity constant (*K*_L_) obtained for Nd^3+^ compared to Y^3+^ indicate a stronger interaction between Nd^3+^ ions and the active surface sites of GO-ATP, demonstrating its preferential adsorption and potential selectivity toward Nd^3+^ over Y^3+^. These findings align with kinetic results and confirm that adsorption is controlled by surface interaction mechanisms with additional contributions from pore diffusion.

#### 3.3.6. Thermodynamic studies

To elucidate the adsorption tendencies and temperature dependence of Nd^3+^ and Y^3+^ ions onto the GO-ATP nanocomposite, thermodynamic parameters were evaluated using the Van't Hoff method as summarized in Table 3.^43^ The calculated values of enthalpy change (Δ*H*°), entropy change (Δ*S*°), and Gibbs free energy change (Δ*G*°) are summarized in Table 5. Additionally, Fig. 12(g) displays the Van't Hoff plots of −ln *K*_L_*versus* 1/*T*, constructed using Langmuir constants at four different temperatures (298–328 K).^26,44^

**Table 5:** Thermodynamic parameters for adsorption of Nd and Y GO-ATP at different temperatures

Adsorbents	Temp. (Kelvin)	Thermodynamic parameters			
		Δ*H*° (kJ mol^−1^)	Δ*S*° (kJ mol^−1^ K^−1^)	*T*Δ*S*° (kJ mol^−1^)	Δ*G*° (kJ mol^−1^)
Nd	298	76.63	0.323	96.25367	−19.6277
	308			99.48366	−22.8577
	318			102.7137	−26.0877
	328			105.9436	−29.3177
Y	298	41.23	0.195	58.0495	−16.8229
	308			59.9975	−18.7709
	318			61.9455	−20.7188
	328			63.8934	−22.6668

The positive values of enthalpy change (Δ*H*°) for Nd^3+^ (76.63 kJ mol^−1^) and Y^3+^ (41.23 kJ mol^−1^) confirm the endothermic nature of the adsorption process. The higher Δ*H*° value for Nd^3+^ indicates stronger interaction with the GO-ATP surface, likely due to enhanced coordination with phosphate and oxygen containing functional groups. The positive entropy change values (Δ*S*° = 0.323 and 0.195 kJ mol^−1^ K^−1^ for Nd^3+^ and Y^3+^, respectively) suggest increased randomness at the solid–liquid interface during adsorption. This behavior can be attributed to the release of structured water molecules (desolvation) and structural rearrangement upon complex formation. The Gibbs free energy change (Δ*G*°) values were negative across all studied temperatures, confirming the spontaneous nature of adsorption. For Nd^3+^, Δ*G*° decreased from −19.63 to −29.32 kJ mol^−1^ with increasing temperature (298–328 K), while for Y^3+^, Δ*G*° ranged from −16.82 to −22.67 kJ mol^−1^, indicating enhanced adsorption favorability at elevated temperatures.

Notably, the increasing contribution of the *T*Δ*S*° term with temperature (reaching 105.94 kJ mol^−1^ for Nd^3+^ and 63.89 kJ mol^−1^ for Y^3+^ at 328 K) demonstrates that the adsorption process is predominantly entropy-driven. This indicates that the overall driving force arises from increased disorder at the solid–liquid interface, primarily due to desolvation and inner-sphere complex formation between REE ions and ATP functional groups. The linearity of the Van't Hoff plots further supports the consistency of the thermodynamic parameters, with steeper slopes observed for Nd^3+^ reflecting its higher Δ*H*° value. The higher intercept values indicate stronger equilibrium affinity (*K*_L_) at elevated temperatures. These findings, in conjunction with kinetic and isotherm analyses, suggest that Nd^3+^ adsorption follows a predominantly chemisorption mechanism, whereas Y^3+^ exhibits a mixed physico-chemical adsorption behavior. Overall, the thermodynamic results confirm the stronger affinity of GO-ATP toward Nd^3+^ compared to Y^3+^ and demonstrate that adsorption becomes increasingly favorable with temperature.

The experimentally determined thermodynamic parameters further support the occurrence of a spontaneous and coordination driven adsorption process between REE ions and the functional groups of the GO-ATP adsorbent. Although advanced theoretical approaches such as density functional theory (DFT) calculations and Born Haber type energetic analyses could provide deeper molecular level insight into adsorption energetics and binding mechanisms, such computational investigations were beyond the scope of the present experimental study. Nevertheless, the integration of experimental thermodynamic analysis with computational modeling represents an important direction for future research.

#### 3.3.7. Effect of solid to liquid ratio

**Fig. 13:**
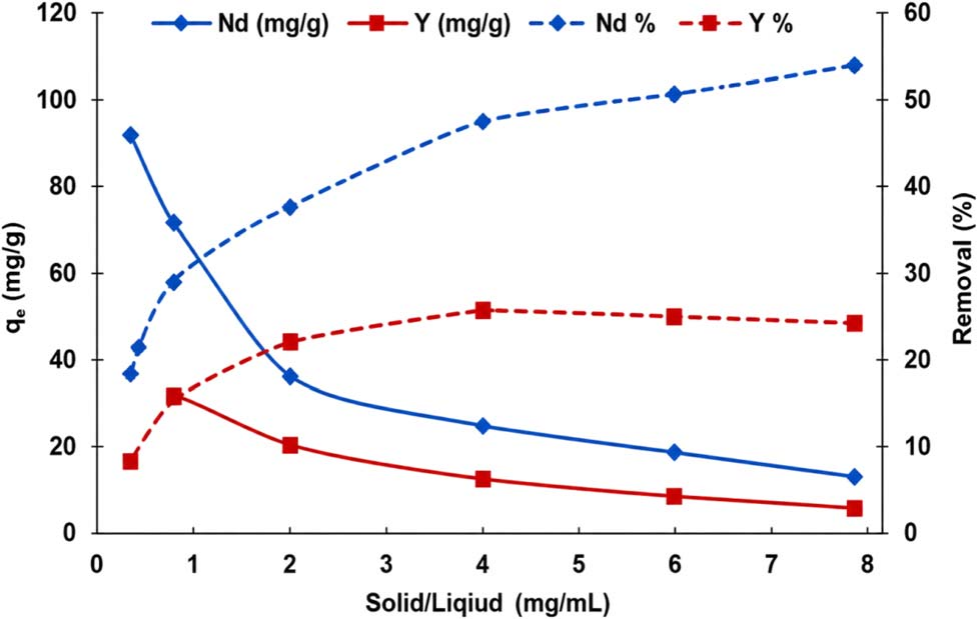
The effect of adsorbent dose on adsorption capacity and percentage removal.

The effect of the solid to liquid (S/L) ratio on the adsorption performance of GO-ATP for Nd^3+^ and Y^3+^ reveals contrasting trends between adsorption capacity and removal efficiency is depicted in Fig. 13. As the S/L ratio increases from 0.25 to 8 mg mL^−1^, the adsorption capacities (*q*_e_) of both ions decline significantly. For Nd^3+^, *q*_e_ decreases from approximately 94 mg g^−1^ to below 15 mg g^−1^, while for Y^3+^, it drops from 44 mg g^−1^ to less than 10 mg g^−1^. This decrease in capacity is attributed to the saturation of adsorption sites and potential agglomeration of the adsorbent particles, which limits access to active binding regions and reduces the surface area available per unit mass of adsorbent. Conversely, the removal efficiency (%) increases with rising S/L ratios. Nd^3+^ removal improves steadily, reaching over 50%, while Y^3+^ removal plateaus around 26% at higher dosages. This improvement is due to the increased total number of adsorption sites, allowing more metal ions to be captured despite lower per gram adsorption efficiency. Notably, Nd^3+^ consistently outperforms Y^3+^ in both metrics, reinforcing GO-ATP higher affinity and selectivity toward neodymium under varying operational conditions.

#### 3.3.8. Desorption studies

**Fig. 14:**
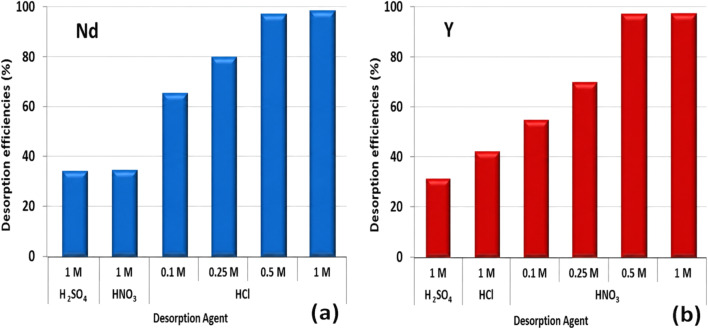
The effect of desorption of Nd (a) and Y (b) using desorption agent.

Desorption experiments were conducted to evaluate the reusability and recovery efficiency of GO-ATP for Nd^3+^ and Y^3+^ ions using various acidic eluents at different concentrations. The results, presented in Fig. 14(a and b), revealed that desorption efficiency varied significantly depending on the type and concentration of the desorbing agent. For Nd^3+^, HCl proved to be the most effective desorbing agent. The desorption efficiency increased with HCl concentration, reaching a maximum of 98.9% at 1.0 M. In comparison, 1.0 M HNO_3_ and H_2_SO_4_ resulted in 46% and 35% desorption efficiencies, respectively. The superior performance of HCl can be attributed to the strong complexation tendency of Nd^3+^ with chloride ions, which form stable soluble Nd–Cl complexes and thereby weaken Nd-phosphate interactions on the GO-ATP surface. Additionally, the relatively larger ionic radius of Nd^3+^ lowers its binding strength to phosphate groups, making it more easily displaced by Cl^−^ ions. Conversely, for Y^3+^, HNO_3_ was the most efficient desorbing agent, achieving 97.3% desorption at 1.0 M. In contrast, 1.0 M HCl yielded only 43% desorption, and 1.0 M H_2_SO_4_ achieved the lowest desorption at 33%. The superior performance of nitric acid for Y^3+^ may be attributed to its stronger oxidative nature and better protonation ability, which enhances the replacement of adsorbed Y^3+^ ions. These findings suggest that desorption behavior is ion specific and should be considered when designing regeneration protocols for GO-ATP-based sorbents. Notably, the distinct desorption behaviors observed for Nd^3+^ and Y^3+^ indicate the feasibility of selective sequential recovery. The preferential desorption of Nd^3+^ using HCl and Y^3+^ using HNO_3_ suggests that stepwise regeneration strategies can be designed to achieve controlled and selective ion recovery. This behavior is governed by differences in coordination strength and hydration characteristics between the two ions. Such findings highlight the potential of the GO-ATP system not only for efficient adsorption but also for practical separation of rare earth elements in hydrometallurgical processes.

#### 3.3.9. Comparison of adsorbent performance

To contextualize the performance of the proposed GO-ATP nanocomposite, Table 6 provides a comparison of the adsorption capacities of various adsorbents for Nd^3+^ and Y^3+^ reported in the literature.

**Table 6:** Comparison of maximum adsorption capacities (*q*_max_) for Nd^3+^ and Y^3+^ using different adsorbents

Adsorbent material	Target REE	Maximum adsorption capacity (*Q*_max_, mg g^−1^)	Reference
GO-TAPA	Nd(iii)	20.6	17
	Y(iii)	10.52	
Magnetic chitosan & graphene oxide (MCh & GO)	Nd(iii)	56.6	13
Nano-MgO/Ca-alginate beads	Nd(iii)	7.85	18
	Y(iii)	5.60	
Fumarated polystyrene microspheres	Nd(iii)	39.69	45
Commercial activated carbon	Nd(iii)	36.65	46
Thiourea modified Amberlite XAD7	Nd(iii)	74.3	47
CS-MF	Nd(iii)	44.29	48
PAM-activated carbon composite	Nd(iii)	9.88	49
EDTA-AC	Nd(iii)	71.4	50
EDTA-chitosan	Nd(iii)	74	51
DTPA-chitosan–silica	Nd(iii)	38.9	52
Alginate-TMOS	Nd(iii)	53.4	53
DETA functionalized chitosan nanoparticles	Nd(iii)	54.8	54
P-functionalized silica	Nd(iii)	73.6	55
GO-ATP nanocomposite	Nd(iii)	96.1538	This work
	Y(iii)	58.139	This work

#### 3.3.10. Application to real raffinate solution

**Fig. 15:**
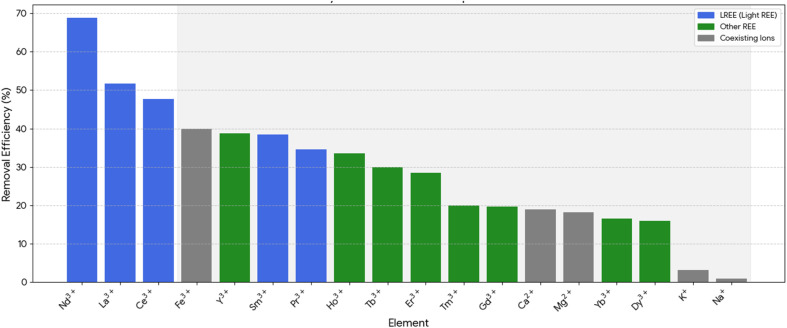
Removal efficiency of GO-ATP composite toward rare earth elements (REEs) and coexisting ions from a real raffinate solution under optimized adsorption conditions.

The practical applicability of the GO-ATP composite was evaluated using a real raffinate solution obtained from the hydrometallurgical processing of Egyptian monazite ore. The raffinate represents a complex industrial leachate composed of light and heavy rare earth elements (REEs) along with coexisting alkaline and transition metal ions originating from gangue minerals and process reagents Table 7. GO-ATP demonstrated high selectivity toward light rare earth elements (LREEs), achieving removal efficiencies of 68.80% for Nd^3+^, 51.68% for La^3+^, and 47.76% for Ce^3+^. In contrast, the adsorption of heavy rare earth elements (HREEs) such as Gd^3+^, Tb^3+^, Ho^3+^, and Er^3+^ was relatively lower (19–34%). This preference for LREEs can be attributed to their larger ionic radii and lower hydration energies, which facilitate diffusion and coordination onto reactive surface sites. Moderate adsorption was observed for Fe^3+^ (39.98%), whereas alkaline and alkaline earth metals such as Na^+^, K^+^, Ca^2+^, and Mg^2+^ exhibited minimal uptake (<20%), confirming the strong affinity of GO-ATP toward multivalent cations, Fig. 15.

**Table 7:** Raffinate composition with initial and final concentrations and calculated removal efficiencies

Element	Initial concentration, *C*_i_ (mg L^−1^)	Final concentration, *C*_f_ (mg L^−1^)	Removal efficiency (%)
Nd^3+^	140	43.7	68.8
La^3+^	270	130.45	51.68
Ce^3+^	110	57.46	47.76
Gd^3+^	11	8.84	19.63
Ho^3+^	5.57	3.7	33.5
Pr^3+^	35.79	23.38	34.6
Sm^3+^	22	13.54	38.4
Tb^3+^	20	14	30
Y^3+^	6	4.89	38.8
Dy^3+^	2	1.68	16
Er^3+^	0.14	0.1	28.5
Tm^3+^	0.2	0.16	20
Yb^3+^	0.06	0.05	16.6
Ca^2+^	12.21	9.89	19
Fe^3+^	36.66	20	39.98
Mg^2+^	100	81.87	18.13
Na^+^	141.0	139.67	0.94
K^+^	35.0	33.9	3.1

This selective behavior is attributed to chemisorption-driven interactions between REE ions and the oxygen-containing functional groups (–PO_4_^3−^, –OH, –COOH) introduced by ATP. Adsorption proceeds *via* inner-sphere surface complexation and ion exchange, where REE ions replace surface-bound protons or water molecules. According to the Hard and Soft Acid–Base (HSAB) theory, REE^3+^ ions (hard acids) exhibit strong binding affinity toward phosphate and hydroxyl groups (hard bases), forming stable coordination complexes. The negligible uptake of monovalent ions suggests that electrostatic attraction alone does not govern the process. Instead, adsorption is dominated by specific ligand coordination, consistent with the kinetic and mechanistic findings. These results confirm the capability of GO-ATP to treat complex hydrometallurgical effluents while selectively recovering strategically important REEs, demonstrating its potential for large scale application in REE extraction and recycling industries.

## 4. Conclusion

Adenosine triphosphate-functionalized graphene oxide (GO-ATP) was successfully synthesized and evaluated as a novel nanocomposite adsorbent for the efficient recovery of Nd^3+^ and Y^3+^ ions from aqueous media. Physicochemical characterization (FTIR, XRD, SEM/EDX, TEM and BET) confirmed the successful functionalization of graphene oxide with ATP, resulting in enhanced surface functionality despite a reduction in specific surface area. Adsorption kinetics followed a pseudo-second-order model, indicating that chemisorption is the dominant rate controlling mechanism. Equilibrium data were well described by the Langmuir isotherm, suggesting monolayer adsorption with maximum capacities of 96.15 mg g^−1^ for Nd^3+^ and 58.10 mg g^−1^ for Y^3+^. Thermodynamic analysis revealed positive Δ*H*° values, confirming the endothermic nature of adsorption, while negative Δ*G*° values indicated spontaneous uptake over the studied temperature range. Notably, the increasing contribution of the *T*Δ*S*° term with temperature demonstrates that the adsorption process is predominantly entropy-driven, reflecting increased disorder at the solid–liquid interface due to desolvation and inner-sphere complex formation between REE ions and ATP functional groups.

The GO-ATP composite also exhibited excellent reusability, with high desorption efficiencies achieved using mild acidic eluents. Importantly, the distinct desorption behavior of Nd^3+^ and Y^3+^ enables selective sequential recovery, highlighting the potential of GO-ATP not only for adsorption but also for practical separation of rare earth elements. The stronger interaction of Nd^3+^ with the adsorbent surface, compared to Y^3+^, is attributed to more favorable coordination with phosphate groups, as supported by spectroscopic analysis and thermodynamic parameters. Overall, the GO-ATP nanocomposite represents a promising and sustainable platform for selective REE recovery from contaminated water and industrial effluents. Its enhanced adsorption performance, regeneration capability, and potential for controlled separation make it a valuable material for advanced wastewater treatment and resource recovery applications. This work provides a mechanistically informed approach for designing biofunctionalized adsorbents tailored for selective rare earth element separation.

## Conflicts of interest

The authors declare that they have no known competing financial interests or personal relationships that could have appeared to influence the work reported in this paper.

## Data Availability

The data supporting the findings of this study are available within the article. Additional raw experimental data are available from the corresponding author upon reasonable request. The additional data consist of original characterization and adsorption datasets generated during the study and can be shared by the corresponding author upon reasonable request.
